# Tissue Engineering; Current Status & Futuristic Scope

**DOI:** 10.25122/jml-2019-0032

**Published:** 2019

**Authors:** Preeti Sharma, Pradeep Kumar, Rachna Sharma, Vijaya Dhar Bhatt, PS Dhot

**Affiliations:** 1. Department of Biochemistry, Santosh Medical College and Hospital (Santosh University), Ghaziabad, UP, India; 2. Department of Biochemistry, TSM Medical College and Hospital, Lucknow, UP, India; 3. Department of Conservative Dentistry and Endodontics, Santosh Dental College and Hospital, Ghaziabad, UP, India; 4. Department of Pathology, Santosh Medical College and Hospital, Ghaziabad, UP, India

**Keywords:** in born error, tissue engeneering, neonates

## Abstract

Almost 30 years have passed since the term ‘tissue engineering’ was created to represent a new concept that focuses on the regeneration of neotissues from cells with the support of biomaterials and growth factors. This interdisciplinary engineering has attracted much attention as a new therapeutic means that may overcome the drawbacks involved in the current artificial organs and organ transplantation that have also been aiming at replacing lost or severely damaged tissues or organs. However, the tissues regenerated by tissue engineering and widely applied to patients are still minimal, including skin, bone, cartilage, capillary, and periodontal tissues. What are the reasons for such slow advances in clinical applications of tissue engineering? This article gives a brief overview of the current state of tissue engineering, covering the fundamentals and applications. The fundamentals of tissue engineering involve cell sources, scaffolds for cell expansion and differentiation, as well as carriers for growth factors. Animal and human trials are a major part of the applications. Based on these results, some critical problems to be resolved for the advances of tissue engineering are addressed from the engineering point of view, emphasizing the close collaboration between medical doctors and biomaterials scientists.

## Introduction

A newer concept named ‘tissue engineering’ came to light almost 30 years ago, the main focus of which was neotissue regeneration. With the help of biological tools in the form of biomaterials and growth factors, engineering of tissues technique emerged as a therapeutic means to treat many of the challenging medical conditions. Though still in its grooming stage, tissue engineering is able to meet the risks involved in organ transplantation and artificial organ implants. However, with a limited scope of the tissues, generated from this process, there is a lot to be done in the field in order to expand its clinical applications in service of mankind. The current review addresses many of the issues and challenges in the field.

Tissue engineering (TE) is the most exciting and rapidly growing area in the field of biomedical engineering. Despite recent technological advancement, thousands of people die every year due to lack of an organ donor or any other efficient substitute for the organ. A branch of regenerative medicine, tissue engineering is nowadays emerging as a means to replace diseased or damaged organs by artificially constructing tissues or organs in vitro and transplanted in vivo. The field of TE is a combination of various other branches of biology including cell biology, material science, chemistry, molecular biology, engineering, and medicine. Two stalwarts, Langer and Vacanti, introduced for the first time in 1993, the term ‘tissue engineering’ [1]. It is the field concerned with biological substitutes that maintain, improve, or restore tissue functions in order to cope up with the problem related to tissue damage. In the current scenario organ transplant, mechanical devices, or surgical reconstruction are prevalent as a substitute for tissue damage [2–3]. No doubt, these techniques have saved the lives of hundreds of patients, but parallelly, they generate several complications as well [4]. Mechanical devices do not accomplish all the functions performed by natural tissues and are not very effective at controlling the progressive deterioration of the patient’s condition [5]. The lack of donor to cover worldwide demand, as well as tissue rejection, is the major hurdles in the way to organ transplantation [6]. Consequences of surgical reconstruction are not always positive and lead to a long-term complaint [7]. TE has emerged as a solution to the problem associated with tissue damage with the development of in vitro tissues to repair in vivo damage. The engineered tissues obtained by TE technique, are of great help to study human physiology and regenerative medicine [8]. The term ‘regenerative medicine’ came into existence when two research groups from the USA have jointly developed human embryonic stem cells and embryonic germ cell lines in 1998 [9]. TE is an emerging technology that uses the combination of cells, engineering methods and materials and suitable biochemical and physicochemical factors to improve or replace biological functions, meant to reconstruct damaged or diseased organs and tissues in vitro and transplantation in vivo to recover lost or malfunctioned organ or tissue [1]. Well, growth and development of cells take place in well-organized three-dimensional extracellular matrix (ECM) surrounded by other cells, and cell-cell and cell-ECM interactions decide upon the fate of the cell for its proliferation or differentiation, apoptosis or invasion [7]. The two-dimensional culture medium is not able to provide natural in vivo environment of cellular communication, gene and protein expression, or diffusion of various soluble molecules [10]. Animal models, as a substitute for human testing, provide insufficient information; they fail to capture the essential facts of human responses, are very costly and lead to ethical controversies. So here comes the importance of TE as a substitute to animal models or culture, provide natural in vivo environment.

### Tissue Engineering; What is the Need?

There are many clinical conditions warrant for the use of engineered tissues or organ. Like in conditions of congenital abnormalities, tissue reconstruction is needed. Following any disease or severe injury, most of the tissues are not able to regenerate or regenerate to a minimal extent, like bone and skin. Also, for organ transplantation, there is a scarcity of donor tissues. As a mean of persuasion of the body to heal itself, tissue engineering technique involves the regeneration of new tissue to replace the one which has become diseased or injured [1,2]. As human beings, there is a limited capacity of tissue regeneration under minimal circumstances. Repair of bone fractures and injured skin are a few examples of tissue regeneration in our body.

The essence of tissue engineering is that those cells capable of initiating and sustaining the regeneration process are ‘switched on’, perhaps through growth factors or genes, so that they generate new functional tissue of the required variety [11]. This may be achieved with the help of a scaffold or matrix to guide the geometrical or architectural shape of the new tissue and may take place on a customized basis at the site of the injury in an individual patient or on a more industrial scale in an ex vivo bioreactor, where the resulting tissue construct is re-implanted into the patient [12]. The field of tissue engineering is a promising one, emerged as a technique to promote, direct and induce the innate capacity of tissues for regeneration and to assist in regaining function and shape, where the chances of natural healing are not possible [1]. In the past few years, a number of advances have been achieved in the regeneration and development of bone, cartilage, heart, pancreas and vasculature, dental implants and other organs [13]. Transforming the knowledge of human physiology and pathophysiology, the TE has emerged as a valuable tool in the field of biomedical research.

### Various challenges to face

This interdisciplinary engineering technique has attracted much attention as a new therapeutic means that may overcome the drawbacks involved in the current artificial organs and organ transplantation that have also been aiming at replacing lost or severely damaged tissues or organs. However, tissue regeneration by TE technique is very less in amount and cannot be widely applied to the patients. In the current paradigm, there has been a very slow and insidious advancement in the clinical applications of TE [14]. Also, the reported clinical trials are limited [15], though a tremendous amount of basic research studies have been done in this direction. TE technique requires three key elements: cells, scaffold, and growth factors [1, 2]. Besides all these, many factors are critical to tissue engineering, such as cell source, construction of scaffold, cell seeding and culture environment, matrix preparation, mechanical characteristics of the cell, scaffold construct and animal models [11].

The types of cell sources enormously influence TE. Autologous (patient’s cells), allogenic (other than patient’s cells) or xenogeneic (derived from animal cells) cells resources are the three significant types considered in TE [11]. Out of these, the xenogeneic cells are considered the least safe as there have been reports of the presence of porcine endogenous retrovirus in pigs [15]. Though safe for TE, autologous cells showed difficulty in harvesting sufficiently in cases of aged patients or severely ill ones [11]. Another drawback with cardiac cells is their harvesting in a poor amount in the patient that suffered from myocardial infarction [16]. In cases of poor harvesting of cells, their expansion is mediated by the method of culturing [16]. However, for this process, only clean cell processing centers are recommended in order to avoid any chance of contamination [16]. Also, the process is lengthy and time-consuming. There have been reports on the infection of fetal calf serum, which is frequently used for cell culturing [11]. Allogenic cells are best suited for skin tissue engineering owing to the powerful secretion of growth factors, but xenogeneic feeder cells used for engineering epidermal tissues from keratinocytes (because of high growth activity), showed a high risk of viral infection [17]. In cases of regeneration and development of large-sized tissues and organs in a three-dimensional manner, support in the form of a scaffold, template or artificial extracellular matrix (ECM) is needed to assist proliferation, differentiation, and bioengineering of cells [14]. ‘Cell therapy’ and ‘tissue engineering’ are two essential terms related to the development and growth of cells and tissues via regenerative medicine [1]. For tissue engineering purposes scaffold should meet some requirements. A scaffold must be sufficiently porous for proper nutrient supply; also, neovasculature formation needs these micropores as well [11]. Waste transport is also possible via these micropores, which are essential for cell survival. However, a significant issue related to the scaffold is the neovascularisation, which is essential for the supply of oxygen and nutrients to the cells in the construct [14]. In case of in vitro tissue engineering, it is impossible to expect vascularisation in the construct [11]. In contrast, neovascularisation is possible in vivo tissue engineering if proper stimuli and conditions are availed [11]. A vast range of growth factor proteins plays a vital role in cell growth, differentiation, and proliferation [14]. These may be autocrine or paracrine released endogenously. Nowadays, the growth factors much in use are bone morphogenic proteins (BMP) [18], basic fibroblast growth factors ( bFGF, bFGF-2) [19], vascular epithelial growth factor (VEGF) [20] and transforming growth factor-beta (TGF-β) [21]. Addition of growth factors (GFs) to the cell scaffold construct is essential for tissue regeneration as they have got the remarkable capability to regenerate tissues [14]. The most critical issue related to GFs is their delivery to the site of action, but bulking injection to the site of regeneration is not an effective mean [11]. So far, three important ways of GFs delivery have been developed: the use of DNA plasmids encoded with the gene of the desired GF, introducing a gene encoding a particular GF into a specific cell type using a vector and implantation of GF proteins directly into the cell via some carriers [14].

GFs-release kinetics is also decided upon by a carrier carrying GF to the site. Generally, a single GF is used for engineering of a single tissue. GFs have been found to induce neovascularization, providing an adequate supply of nutrients to the developing tissues [21]. However, the use of GFs is not very cost-effective, hindering its applicability in TE. Another hurdle coming way to TE is the availability of animal models [11], which cannot be frequently used majorly due to ethical constraints. In fact, many times, the nude mouse model is tiny to avoid any immune rejection of cell scaffold construct implanted [11] whereas large animal models are needed to mimic clinical situations in human beings. In order to assess the scope of tissue engineering of bones and articular cartilages, the evaluation of biochemical properties of normal bone and cartilage and their functional assessment is critical [22]. The technique to measure the above characteristics of normal, diseased, and bioengineered bone in vivo is fundamental, but it is still under developmental stage [22]. Similarly, although many in vitro and in vivo experiments have been performed on cardiovascular diseases, which represent the most abundant cause of mortality [23], long-term studies are still needed to be done in bigger animals before bioengineered blood vessel valves, and heart tissues become commercially available in the market.

### Major Advances in Tissue Engineering to Meet Various Challenges

Considering the progress in tissue culture, preparation of scaffold techniques, scaffold 3D printing, and use of animal models for in vivo applications, the TE technique is advancing at great pace to overcome the challenges and major problems coming its way regarding clinical application [24–26].

Due to the limited supply of autologous stem cells for proliferation in tissue culture, enormous interest arose all through the world on stem cell plasticity in tissue culture. For this purpose, multi- or pluripotent stem cells were isolated from various regions of the body, such as interstitial or circulating cells, mesenchymal stem cells (MSC), and so forth [27]. MSCs are of so much importance and have been used for cartilage repair as microencapsulation of the cells increases their longevity and efficacy [28]. Human embryonic stem cells (hESCs) derived from the inner core of blastocytes (via in vitro fertilization) were also able to generate many cell lineages in tissue culture [29]. Besides all these, the development of induced pluripotent stem cells (iPSC) has been proved to be real advancement in various in vitro studies [30].

With regard to culture conditions for cell regeneration, many improved strategies have been made in order to increase the efficacy and output of the system. Some stem cells were found to be activated under physiological hypoxic conditions as has been found for muscle stem cells in contrast to routine culture conditions of high oxygen level (20%). Simple optimization in culture conditions for various parameters like oxygen supply, growth factors, extracellular matrix, to closely reach the living cell conditions, can significantly improve cellular response in the TE processes [31]. Recently, the major role played by non-coding RNAs (ncRNAs) in the molecular regulation of cell biology and tissue synthesis has also been identified, and these ncRNAs are found to be new targets for in vitro and in vivo tissue growth [32].

Many ways have been optimized with regard to scaffold preparation, material consistency, vascularisation, and so forth. A 3D extracellular matrix secreted by residing tissues is like a scaffold, helps in tissue synthesis via various molecular signals in the course of regeneration with requisite molecular composition, physiological activities, and mechanical strength [33]. The native ECM for optimal growth and development of tissues can be obtained by careful digestion of living cells while retaining the maximum amount of ECF from the specific tissue site. Such kind of successful transplantation of human acellular scaffold has been clinically done for various tissues. But with the simultaneous threat of infection and immunogenicity with native ECM derived from non-human mammalian sources led to an interest in synthetic biomaterials for 3D scaffold construction [34]. These artificial scaffolds consisting of collagen fibronectin, elastin are often combined with various source generated stem cells, such as human-induced pluripotent stem cells (hiPSCs). Biodegradable silk fibroin protein, alginate (seaweed polysaccharide) and apple cellulose have been used for scaffold preparation. Hydrogel and polyacrylamide are widely used synthetic polymers for 3D scaffold preparation [35]. In order to increase the vascular supply, thick implantable viable tissue constructs have been the focus of research [36]. Combination of endothelial cell sheets and muscle precursors have been used to build vascularised heart muscles [37]. These and a number of other approaches need careful validation before clinical application. The 3D scaffold printing technique with desired cells for tissue preparation has completely revolutionized the TE technique by advancing the construction of biomimetic living tissues [38]. These kinds of strategies will further lead to the synthesis of vascularised 3D soft organs to meet the organ transplant crisis.

## Conclusion

The field of tissue engineering holds many promises to meet various challenges with newer innovative developments and shall shortly bring many advances to apply to human beings successfully. The TE involving human stem cells merging with various approaches for bioscaffolds generation offers new avenues for basic research in the field of various scientific fields including biology, physiology, HTS (high-throughput screening) for the pharmaceutical industry, pharmacokinetics and others as well. We have seen various examples of clinically successful implantations of acellular bioscaffold with host cell growth, and in the near future, its applications are more likely to spread rapidly in the service of mankind. Significant challenges to TE regarding in vivo applications are the low survival rate of the donors or autologous cells in vivo that are initially grown in tissue culture, and improper vascularisation of the implanted tissue/organ. However, the future appears to be bright. The use of acellular scaffolds, providing shelter to host cells has expanded the clinical application of TE many folds. The 3D printing of scaffolds with desired cells for tissue preparation has wholly revolutionized the TE technique by advancing the construction of biomimetic living tissues. While thick implantable viable tissue is being used to overcome the vascularisation issue, a very balanced and keen optimization and evaluation of various newer techniques along with ethical considerations are required to promote its potential clinical applications.

Clinical purposes acellular scaffolds that provide a new home for host cells are already being used in various situations and are likely to have expanded applications. The primary clinical obstacles relate to problems with the transfer of living cells from the culture conditions into the human body; this applies to many isolated cells, tissue constructs and artificially engineered organs. Additionally, the vascularisation of implanted tissues/organs at the human scale is very challenging. While the rapid and creative advances in tissue bioengineering hold great promise for the future of regenerative medicine, balanced and critical evaluation of these new technologies, including robust ethical discussions, is required for realistic consideration and promotion of potential clinical applications.

## Conflict of Interest

The authors confirm that there are no conflicts of interest.
